# Endogenous Testosterone and Exogenous Oxytocin Modulate Attentional Processing of Infant Faces

**DOI:** 10.1371/journal.pone.0166617

**Published:** 2016-11-18

**Authors:** Sarah K. C. Holtfrerich, Katharina A. Schwarz, Christian Sprenger, Luise Reimers, Esther K. Diekhof

**Affiliations:** 1 University of Hamburg, Biocenter Grindel and Zoological Museum, Institute for Human Biology, Neuroendocrinology Unit, Hamburg, Germany; 2 Department of Systems Neuroscience, University Medical Center Hamburg-Eppendorf, Hamburg, Germany; Centre de neuroscience cognitive, FRANCE

## Abstract

Evidence indicates that hormones modulate the intensity of maternal care. Oxytocin is known for its positive influence on maternal behavior and its important role for childbirth. In contrast, testosterone promotes egocentric choices and reduces empathy. Further, testosterone decreases during parenthood which could be an adaptation to increased parental investment. The present study investigated the interaction between testosterone and oxytocin in attentional control and their influence on attention to baby schema in women. Higher endogenous testosterone was expected to decrease selective attention to child portraits in a face-in-the-crowd-paradigm, while oxytocin was expected to counteract this effect. As predicted, women with higher salivary testosterone were slower in orienting attention to infant targets in the context of adult distractors. Interestingly, reaction times to infant and adult stimuli decreased after oxytocin administration, but only in women with high endogenous testosterone. These results suggest that oxytocin may counteract the adverse effects of testosterone on a central aspect of social behavior and maternal caretaking.

## Introduction

Strong mother-infant interactions are vital for reproductive success [1]. They are modulated by different hormones, for instance the neuropeptide oxytocin, which is particularly known for influencing maternal behavior [2,3]. It not only plays a major role in the induction of labor and lactation (*here* in rats) [4], but also modulates behavior associated with caretaking (e.g., nestbuilding and nursing in mice) [5] and bonding [6]. Additionally, oxytocin administration improves empathy in humans [3,7]. Especially during the nursing period, improved empathy may enhance the mothers’ capacity to understand the nonverbal needs of her offspring [8].

In contrast to oxytocin the androgen testosterone is mainly associated with aggressive and dominant behavior [9–11] and may decrease caretaking, helpfulness [12], and empathy [13–15]. Furthermore, testosterone promotes masculinization and correlates negatively with prosocial behaviors that are important for childrearing (e.g., nurturance and empathy) and maternal attributes [3,9,12] (but also see [16,17] for a different role of testosterone in male-male cooperation during intergroup conflict). During parenthood testosterone levels decrease, which could be an adaptation to facilitate parental investment [18,19]. These previous findings raise the question considering a possible interaction and even opposing roles of testosterone and oxytocin in parental care. First evidence for an interaction between these two hormones in the modulation of behavior comes from a study by Winslow and Insel [20] on the hormonal modulation of sexual and aggressive behavior. The authors reported an increase of these behaviors in male squirrel monkeys after oxytocin administration, but only in dominant males with high plasma testosterone concentrations [20]. Further support for an interaction between testosterone and oxytocin is offered by research on variations in the oxytocin receptor gene that seem to be linked to a common biomarker (2D:4D ratio) to measure prenatal testosterone–but only in male participants [21]. Hence, these results also suggest that the interaction between testosterone and oxytocin is sexually dimorphic. Another study, which investigated hormonal shifts in Tsimané hunters, found that both, endogenous oxytocin and endogenous testosterone, increased after hunting [22]. The authors presumed that the elevated testosterone levels might have resulted from successful hunting while the increase of oxytocin could enhance social salience. Further suggesting an interaction between both hormones was the fact that the percentage of increase of both hormones was strongly correlated [22].

Most interestingly, there exists also initial evidence considering the interacting effects of oxytocin and testosterone on paternal behavior. Weisman et al. [23] found that the administration of 24 IU oxytocin nasal spray versus placebo led to a short-term alteration of salivary testosterone concentration in fathers. The authors observed that paternal behavior like positive arousal, social gaze, and vocal synchrony increased through oxytocin related changes in testosterone concentrations. In addition, lower baseline testosterone levels in those fathers were associated with more optimal paternal behavior [23]. Even though low testosterone levels as well as high oxytocin levels are usually linked to parental behavior [18,19,24–27] there might exist situations that lead to an increase of both hormones in order to modulate behavior (such as hunting-, protection-, or sexual-behavior) [22,23,26,27]. Nevertheless, a hormonal mechanism through interaction between testosterone and oxytocin that increases responsiveness to babies could be an adaptive feature that facilitates caretaking in adults and should thus enhance reproductive success.

One known mechanism, that automatically elicits parental care and thus enhances the survival rate of the offspring, is the baby schema [28–30]. Baby schema is a key stimulus (“Schlüsselreiz”) that automatically elicits caretaking in adults [28]. It is defined as a combination of different child characteristic features like chubby cheeks, a large forehead, and huge eyes [28]. Previous studies revealed that baby schema induces protection behavior [31], motivates adults to speak in “baby language” [32], and initiates caretaking behavior [33,34]. The impact of cuteness perception and baby schema was investigated by a number of studies [29,35–38]. Important to note is that baby schema seems to be a universal stimulus. On the one hand, because it appears in almost every mammal [28], and on the other hand, because the degree of baby schema in human adults or infants, cats and dogs modulates cuteness perception [37,39–41]. Especially for women of reproductive age, newborns are extremely relevant stimuli, because their survival is directly linked to reproductive success. This applies not only to one’s own, but also to other, associated offspring. Alloparental investment is common in humans, because human infants are very costly to raise. The assistance of alloparents could thus act as a benefit for reproductive success [42]. In this case, the stimulus baby schema should not only lead to a positive emotional reaction, but may also provoke prioritized attention as stimuli with high biological relevance are assumed to be processed preferably by the attentional system [43]. If so, adult threatening stimuli (lions and polar bears in this case) should have a processing advantage in the attentional system too, because of their immediate relevance for survival [44].

Several studies suggest differences in the intensity of parental behavior between men and woman (for review see [3]). Cárdenas and colleagues [42] for example, were able to show that even young nulliparous women looked longer at infant faces and fixated the infant faces more often than female or male adult faces, whereas men were only interested in infant faces when these were presented with a male adult face and not with a female adult face. Nevertheless, the research field of the role of reproductive hormones, such as progesterone [45], estradiol [46] and testosterone [23] in parental behavior is still relatively young (for review see: [27] and [47]). Also the role of testosterone in processing baby schema and its interaction with oxytocin remains rather elusive to date (for review see: [3,24,26,27].

The present project had the aim to investigate the effects of testosterone and oxytocin on behavior associated with parental care and to gain further insights regarding a possible interaction between these hormones. For one thing, we wanted to examine to what extent the endogenous testosterone level influences the processing of visual key stimuli (baby schema in different species) in women (Study I). Specifically, we wanted to assess how endogenous testosterone modulates selective attention to these stimuli and their evaluation. Based on previous evidence on the role of testosterone in social cognition, we predicted that in women the endogenous testosterone concentration should negatively correlate with the speed of attentional orienting to pictures of infants. For another thing, we were interested in the interaction between endogenous testosterone and exogenous oxytocin (i.e., intra-nasal administration), since these hormones seem to exert opposite influences on maternal caretaking behavior (Study II) [23,24,27]. For this purpose, we examined the effect of the “bonding hormone” oxytocin on attention to baby schema in women with high versus low endogenous testosterone concentrations.

We expected that oxytocin administration would promote maternal behavior by diverting prioritized attention towards baby faces especially in women with high testosterone concentrations.

## Methods

The present project was divided into two parts. In both studies, participants performed a computer based implicit *face in the crowd* paradigm (i.e., the ‘*target detection task’*). The target detection task was based on the *odd one out* principle. The participant was instructed to select the one stimulus out of four pictures that did not fit in the crowd (e.g., the infant among three adults, or the adult among three infants). Our aim was to gain information about selective attention as represented by the reaction time (RT). As implied by the specific demand of the target detection task, RT should decrease if the attention is focused on the target (an infant or an adult individual): However, if the participant’s attention is focused on the three pictures that were used as distractors (three infant or three adult individuals), RT should increase.

In the first study we examined the universality of the baby schema by using different human and non-human animal species as stimuli. For each species we had two conditions: the high distracting condition (with the adult target and the infants as distractors) and the low distracting condition (with the infant target among three adults).

In a second study we investigated the relevance of the amount of baby schema for attention processes with another target detection paradigm. To this end, we manipulated different pictures of human babies with respect to the degree of their baby schema. We had three different high distracting conditions in this task (adult among three babies with high, neutral or low amount of baby schema) and three different low distracting conditions (baby with high, neutral or low amount of baby schema among three adults).

All participants were women of reproductive age who took hormonal contraception. Informed and written consent was given by each subject and ethical approval was obtained from the ethics committee of the *Ärztekammer Hamburg*.

### Methods Study I

#### Participants of Study I

Twenty healthy women (mean age ± *SD* = 23.95 ± 2.67 years) with normal or corrected to normal vision were recruited to participate in this study. They received 15 Euros for their participation.

#### Stimuli and procedure of Study I

In the first study, we used pictures of humans (female and male), two species of predators (lion and polar bear), two domestic animals (dog and goat) and a non-human primate (orang-utan). Pictures from different species were used as stimuli in order to test for the universality of baby schema (see [43] for a similar procedure). We took the pictures of adult humans out of the FACES database of the Max-Planck-Institute Institute for Human Development, Center for Lifespan Psychology, Berlin, Germany [48]. All other pictures were obtained from a google picture search.

**Fig 1:**
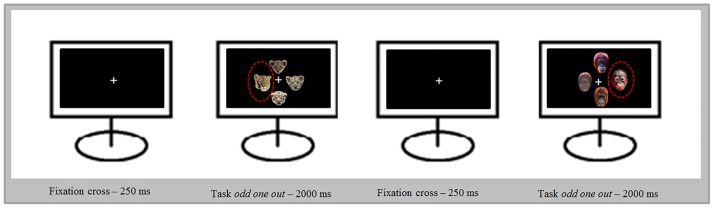
Illustration of two trials out of the *target detection paradigm*.

Using Adobe Photoshop CS5 the faces were cropped from the background and presented on a black square sized 300 pixels. In each trial (duration = 2000 ms), four pictures of the same species were arranged in a cross pattern and presented on a computer monitor. The presented pictures of adult humans and lions were from the same gender. The gender of the other animals was not obvious. The order of the trials was randomized. Subjects were asked to identify the *odd-one-out* on a display of four faces (either an adult target out of three infant distractors or an infant target out of three adult distractors; see Fig 1) via button press, which had to be executed as fast and as accurate as possible. The button positions conformed with the picture positions. During this procedure, the participant’s response time to the targets was measured. The duration between the appearance of the stimulus and the button press was associated with the selective attention the stimulus attracted.

Fig 1 illustrates an example of two trials of the *target detection paradigm*. The paradigm had 392 trials. Each combination was shown 56 times (28 times with the baby target and 28 times with the adult target).

#### Sampling procedure of Study I

Eppendorf tubes (2 ml) for saliva collection were handed to the participants in advance. To determine a proxy of the bioactive testosterone (T) concentration, subjects were instructed to collect five saliva samples in the morning at the day of testing. This was done to control for fluctuating secretion patterns of T. Subjects were asked to provide a saliva sample every 30 minutes starting at normal wake up time. During the sampling procedure (two hours in total) they were not allowed to eat or to consume any other animal source products like, for instance, milk. Furthermore, the time lag between the last consumption of animal source products and saliva sampling had to be at least 12 hours. Smoking was forbidden during the whole sampling procedure. Subjects were allowed to drink water between collection intervals as well as to brush teeth directly after the first sample collection (to avoid blood contamination), but only until five minutes before the sample collection.

Until analysis, saliva samples were frozen at—20°C. Before assaying, saliva samples were thawed and then centrifuged at RCF 604 x g for 5 minutes (i.e., 3000 rpm in a common Eppendorf Minispin centrifuge) to separate saliva from mucus. Following this, an aliquot out of the five samples was prepared by extracting and combining equal volumes (depending on the filling level of the tubes) of the clear and colorless supernatant from each sample. Subsequently, saliva samples were analyzed with a T luminescence immunoassay from IBL (Hamburg, Germany). Each sample was assayed twice and two standards as well as one high and one low control sample were also analyzed on the same assay. Intra- and inter-assay coefficient variances were denoted to range between 1.47–3.01% and 4.04–6.96%, and the formal sensitivity of the assay kit between 1.8 pg/ml and 500 pg/ml T in saliva. Samples were pipetted into the assay plate. The plate was coated with mouse anti-testosterone antibody. Standards and one high and one low control sample were also analyzed on the same assay. Analysis was performed according to the description in the manual and took place in our in-house laboratory.

Salivary measurements provide a reliable and precise method to quantify the bioactive and unbounded T concentration, because the bounded T cannot pass the salivary glands [9]. Moreover, salivary measurements offer a decisive advantage compared to serum measurements in behavioral studies: They are non-invasive and therefore stressless for the participant [49].

#### Data analysis Study I

Data were analyzed with IBM SPSS statistics 19. All data were tested for a significant deviation from normal distribution with the Kolmogorov Smirnov Test. Response times were analyzed with a 2 x 7 (target (adult or infant) x species) repeated measures ANOVA with T concentration as covariate. We used the Greenhouse-Geisser corrected values, if the sphericity assumption was not met. Paired *t*-tests were used for follow-up comparisons.

The mean T concentrations from the aliquot were correlated with response time differences representing the distraction by baby schema in human babies using Pearson correlations. For this purpose, relative RTs were calculated by subtracting the low distracting condition (i.e., infant target and adult distractors) from the high distracting condition with an adult target and three baby faces as distractors. The higher the relative RT the more the participant was distracted by the baby schema. Three participants were excluded from data analysis because of missing T data and one participant was excluded because of problems during data recording. Significances are reported two-tailed if not otherwise indicated and one-tailed in case of clear a priori assumptions.

### Methods Study II

#### Participants Study II

Thirty-eight healthy and nulliparous women were recruited to participate. They had normal or corrected to normal vision. Mean age (± *SD*) averaged 24.12 ± 2.44 years. They received 25 Euros for their participation.

#### Stimuli and procedure of Study II

**Fig 2:**
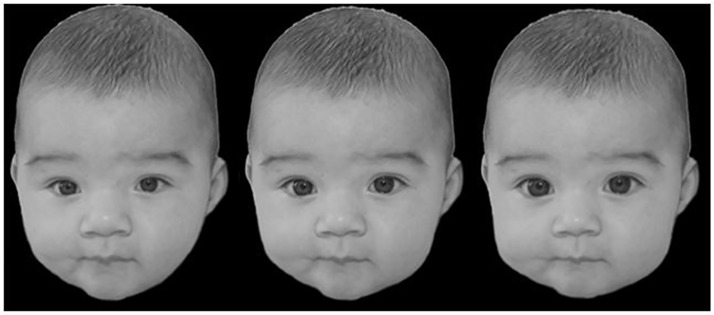
Example for a parametrically manipulated baby. From left to right: low BS condition, unmanipulated baby picture and high BS condition.

To investigate the effects of high habitual T concentrations on selective attentions towards baby faces after oxytocin administration versus placebo administration, we employed a replication of study I, but with a changed stimulus set. On basis of the results of study I, we used only human pictures to investigate the relevance of the amount of baby schema for attention processes. The presented pictures of adult humans were from the same gender. To this end, we parametrically manipulated the pictures of human infants with respect to the amount of baby schema (BS) using Adobe Photoshop CS5. We altered the size of the eyes, mouth, nose and the shape of face, which are the typical features that determine the baby schema after Konrad Lorenz (see [33] for a similar procedure). For an example of the manipulation stimuli see Fig 2.

Prior to our study the pictures of the babies were evaluated by another 36 female participants using a scale from zero to nine (0 =“not cute at all”, 9 =“extra cute”).

For the target detection task we only used pictures, for which the manipulated version with a high amount of baby schema was rated as significantly cuter than the corresponding low baby schema version as shown by Wilcoxon-tests (**Baby A**: *M*_*minus*_ = 6.28, *SD* = 2.26; *M*_*plus*_ = 7.19, *SD* = 2.12; *Z* = -3.334, *p* = 0.001; **Baby B**: *M*_*minus*_ = 4.97, *SD* = 2.26; *M*_*plus*_ = 7.25, *SD* = 2.32; *Z* = -4.711, *p* < 0.001; **Baby C**: *M*_*minus*_ = 5.03, *SD* = 2.47; *M*_*plus*_ = 7.06, *SD* = 1.90; *Z* = -4.460, *p* < 0.001; **Baby D**: *M*_*minus*_ = 5.50, *SD* = 2.51; *M*_*plus*_ = 6.50, *SD* = 2.57; *Z* = -3.105, *p* = 0.002).

The faces were cropped from their original background and presented on a black square.

The pictures of adult humans were taken from the data base FACES from the Max-Blanck-Institute for Human Development, Center for Lifespan Psychology, Berlin, Germany [48]. Infant pictures were obtained from a google picture research.

As in study I, each trial consisted of four pictures that were arranged in a cross pattern around a fixation cross. In each trial either three baby pictures with the same amount of BS (i.e., high BS, low BS, or unmanipulated BS) and one adult target were shown, or three adults in combination with one baby target (high BS, low BS, or unmanipulated BS) were presented (The experimental procedure conformed to study I but the stimuli in this task consisted of human faces only; the infant faces were manipulated in their amount of baby schema). The order of the trials was randomized.

Each trial lasted 2000 ms. Altogether, this paradigm consisted of 240 trials with each condition shown 40 times.

#### Oxytocin administration in Study II

The experiment was double-blind placebo controlled and used a between subjects design. 19 of the female participants were included in the oxytocin group (experimental group) and 19 were included in the placebo group (control group). In the presence of an assistant, participants self-administered a nasal spray. The nasal spray contained either oxytocin or the placebo-control consisting of chlorobutanol-hemihydrat (0.5%) with no active treatment. In total, 24 IU of oxytocin nasal spray (Syntocinon) were administered per participant in the experimental group. It is known from previous studies that 24 IU of intranasal oxytocin are sufficient to increase the oxytocin levels in saliva up to 6 to 10-fold higher than oxytocin levels in the placebo condition 7 hours after administration (for a review see [50]). After administration we waited 45 minutes to continue with the experimental procedure to ensure high treatment efficacy [51].

Former research has shown that the beliefs about the hormonal administration can also influence the behavior of the participant [52]. To control for the participants’ treatment expectations, we pseudo-instructed the participants about the content of the nasal spray. For this purpose the assistant handed an envelope, which included either an ‘oxytocin’ or an ‘empty compound’ label, to the participant. Half of the participants of both the experimental and the placebo group received the information that their nasal spray contained oxytocin. The other half was instructed that their nasal spray contained an inactive substance. The participants were instructed by the assistant not to inform the experimenter about the content of the nasal spray. Hence, the experimenter neither knew which treatment nor which instruction a participant was given. The assistant left the room after the nasal sprays were administered, so that the following questionnaires and experiments were conducted only under the supervision of the experimenter in a double-blind fashion. After the experimental sessions were completed, the participants were asked if they had believed the previous instruction.

#### Sampling procedure of Study II

In this study three saliva samples were collected over one hour directly after waking up. This was done for reason of time economy, since the whole testing procedure (i.e., oxytocin administration and behavioral testing) required another two hours on the same day. However, three samples are sufficient for a representative daily T measure.

#### Data analysis of Study II

Data were analyzed with IBM SPSS statistics 19. The general effects were assessed in a 2 (drug group) x 2 (median split of the testosterone concentration) x 2 (target: adult or infant) x 3 (condition: target or distractor with low BS, unmanipulated BS, or high BS) repeated measures ANOVA.

For exploratory research on the basis of the results of study I, the sample was then divided into two parts via a median split to separate women with high from women with low T concentrations. Response times were then analyzed with a 2 (drug group) x 2 (target: adult or infant) x 3 (condition: target or distractor with low BS, unmanipulated BS, or high BS) repeated measures ANOVA. Greenhouse-Geisser corrected values are reported in cases where the sphericity assumption was not met. Post hoc *t-tests* were used to compare the mean RTs between the control and the experimental group in women with high and low T concentrations and to compare the RTs of low T and high T subjects directly.

For replication of study I, T concentrations were correlated with response time differences representing prioritized attention to baby schema using Pearson correlations. For this purpose, relative RTs were calculated by subtracting the low distracting condition (i.e., infant target and adult distractors) from the high distracting condition (i.e., adult target and three baby faces as distractors). Additionally, we analyzed the relative RTs (representing prioritized attention to baby schema) for the three baby schema conditions separately. Finally, to test for a possible belief effect through pseudo-instruction, the response time differences were analyzed with a one-way ANOVA (for a similar analysis procedure see [52]) including the independent variable pseudo-instruction (i.e. if the participants were instructed accurately or incorrectly). Significances are reported two-tailed if not otherwise indicated and one-tailed in case of clear a priori assumptions.

## Results

### Results of Study I

The RTs were significantly influenced by the species of the presented pictures (*F*_6,102_ = 68.23, *p* < 0.001, *n*_*p*_^*2*^ = 0.801), and the age of the target (adult vs. infant) (*F*_1,17_ = 6.14, *p =* 0.024, *n*_*p*_^*2*^ = 0.265). Furthermore there was a significant interaction between the age and the species of the target (*F*_6,102_ = 3.32, *p* = 0.005, *n*_*p*_^*2*^ = 0.163). We also found a statistical trend for an interaction between target age and T concentration (*F*_1,17_ = 3.77, *p =* 0.069, *n*_*p*_^*2*^ = 0.182).

Post hoc *t*-tests showed that participants required significantly more time to select an adult stimulus when primates (including humans) and goats were presented (all *p value*s < 0.05). We also observed a trend in the same direction in the dog condition (*p* = .0.092, one-tailed).

**Fig 3:**
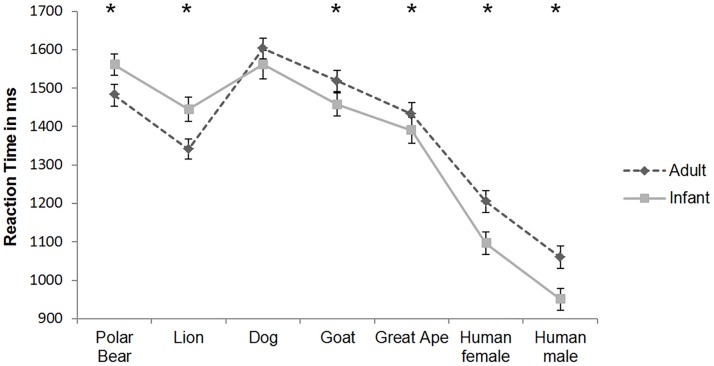
Mean reaction times in ms to select the infant or the adult target (± SEM). * p < 0.05, paired t-test, one-tailed.

In contrast, participants required significantly more time to locate the infant predator (lion or polar bear) among three adult predators. Results of post hoc *t*-tests are presented in Fig 3.

**Fig 4:**
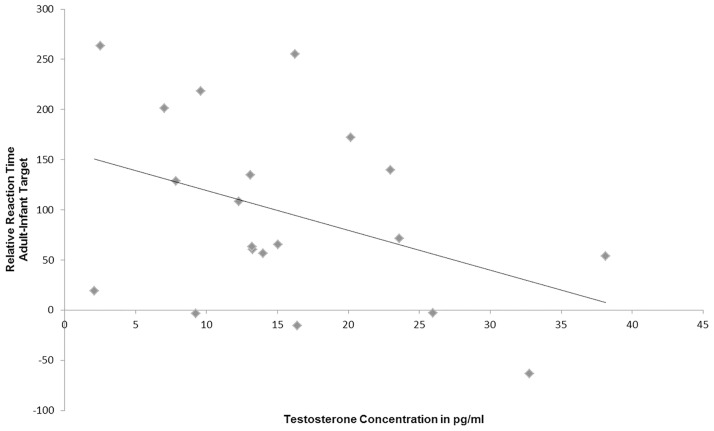
Negative correlation between salivary testosterone concentration and selective attention to human infant portraits (*r* = -0.402, *p* = 0.04, one-tailed).

The mean salivary T concentration of the participants was *M* = 15.77 pg/ml ± *SD* = 9.3 pg/ml. T levels were negatively associated with the distraction by baby schema in human pictures as represented by the relative reaction times (i.e., RTs for adult target minus RTs for adult distractors: *r* = -0.402, *p* = 0.04; one-tailed; see Fig 4). Thus, T concentration in women seems to influence orientation and responding to infant targets.

### Results of Study II

The mean T concentration of the participants was *M* = 18.84 ± *SD* = 16.97 pg/ ml. T concentration did not differ between the medication groups (*M*_*oxytocin group*_ = 15.8 pg/ml ± *SD* 16.42; *M*_*placebo group*_ = 21.88 pg/ml ± *SD* 17.41; *t*_*36*_ = -1.108, *p* = 0.275). To control for so called ‘belief effects’ the participants were pseudo-instructed about the content of the nasal spray, whereby half of the participants were instructed accurately and half of the participants were instructed incorrectly. This was done in light of previous research suggesting that the “folk wisdom” about the effects of the androgen T is partly responsible for behavior of the participant [52]. Interestingly, if the participants thought they got oxytocin, they were significantly slower in orienting attention towards adult targets and the minus baby target condition (p < 0.05). When participants really got oxytocin participants were faster in identifying any target in the minus condition (p < 0.05) and still, with a statistical trend, in the plus condition (p < 0.1). However, in the present study, the pseudo-instruction (if the participant was instructed accurately or incorrectly) had no effect on the distraction by baby schema (e.g. Delta adult target—baby target) (*F*_*1*,37_ = 0.716, *p* = 0.403), only on the reaction times in general, which is important for the following analysis. Therefore this will not be considered in the following analyses, but is a very interesting research topic for further studies.

The general effects were analyzed in a 2 (drug group) x 2 (median split of the testosterone concentration) x 2 (target) x 3 (condition) ANOVA. We found an interaction between target age (adult or baby) and condition (*F*_2,68_ = 6.277, *p* = 0.003, *n*_*p*_^*2*^ = 0.156). There also was a significant effect of the condition (*F*_2,68_ = 5.685, *p* = 0.005, *n*_*p*_^*2*^ = 0.143) and of the target age (adult or infant) (target age: *F*_1,34_ = 25.404, *p* < 0.001, *n*_*p*_^*2*^ = 0.428). The effect of the drug group (*F*_1,34_ = 2.347, *p* = 0.135, *n*_*p*_^*2*^ = 0.065) and the effect of the median split of the testosterone concentration did not reach significance (*F*_1,34_ = 2.270, *p* = 0.141, *n*_*p*_^*2*^ = 0.063). We could not find any further interaction between the drug group or the median split of the testosterone concentration and the other factors.

As T concentrations decrease in parents during parenthood [18,19] and oxytocin leads to increased bonding behavior during parenthood [53], it seems viable that particularly individuals with high endogenous T levels might be affected by oxytocin administration. For exploratory reason and on the basis of the results of study I we tested this hypothesis, by dividing the participants into a high and a low T group (*n* = 19 per group). A 2 x 2 x 3 ANOVA was run to test for effects of target age and the condition of the target or the distractor (low BS, high BS or unmanipulated BS) on the attention behavior in women with low and women with high T concentrations separately. In women with low T concentrations the condition of the target or the distractor affected the participants’ RT to the target stimuli (*F*_2,34_ = 4.59, *p* = 0.017, *n*_*p*_^*2*^ = 0.213). We also found a significant effect of target age (*F*_1,17_ = 6.487, *p* = 0.021, *n*_*p*_^*2*^ = 0.276). We also found a significant main effect of the interaction between target age and condition (*F*_2,34_ = 4.092, *p* = 0.026, *n*_*p*_^*2*^ = 0.194). There was no significant effect or interaction with the other factors of medication in the low T condition.

Interestingly, in women with high T concentrations, there was a significant main effect of medication (*F*_1,17_ = 5.096, *p* = 0.037, *n*_*p*_^*2*^ = 0.231). However, the condition of the target or the distractor did not reach significance anymore (*F*_1,34_ = 1.969, *p* = 0.15, *n*_*p*_^*2*^ = 0.104) and the interaction between target age and condition only reached statistical trend level (*F*_1,34_ = 2.461, *p* = 0.1, *n*_*p*_^*2*^ = 0.126). Nonetheless, there was an effect of the factor target age in the high T condition (*F*_1,17_ = 24.067, *p* < 0.001, *n*_*p*_^*2*^ = 0.586).

**Fig 5:**
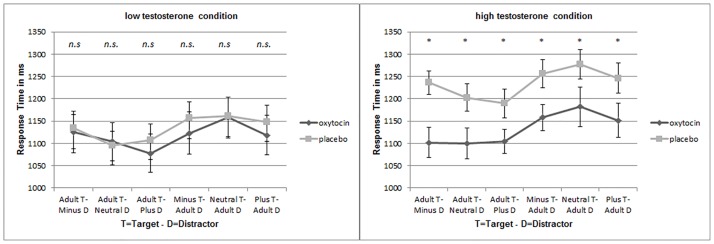
In both figures, the mean reaction times to select an adult or an infant target are shown for both administration conditions (oxytocin or placebo) (± SEM). The reaction times to select an infant target are shown for the three baby schema conditions: minus = low BS, neutral = unmanipulated BS, plus = high BS. On the left side you can see the results for women with low T concentrations (n_lowT_ = 19) and on the right side you can see the results for women with high T concentrations (n_highT_ = 19). Not significant = n.s., * = *p* < 0.05, t-test, one-tailed.

T-tests were used to compare the mean RTs between the experimental and control group (i.e., oxytocin vs. placebo). We were especially interested in the effects of oxytocin on attention to baby schema in women with high versus low endogenous T concentrations. The participants’ mean RTs to adult or infant targets did not differ between the oxytocin and placebo treatment groups in women with low T concentrations (for low T women see Fig 5 left side). However, women with high T concentrations needed more time to locate the target when they were in the placebo treated group (irrespective of target age and amount of baby schema). Interestingly, RTs of high T women that were treated with oxytocin aligned with the low T group (there was no significant difference between response times of high T women that were treated with oxytocin and response times of the low T women in the oxytocin or placebo condition) (see Fig 5 right side). The RTs of the low and the high T subjects differed significantly in all three baby target conditions (high BS: *t*_36_ = -1.994, *p* = 0.026; neutral BS: *t*_36_ = -1.952, *p* = 0.029; low BS: *t*_36_ = -2.086, *p* = 0.022, *t*-test; one-tailed) and in the adult target condition with the plus morphed distractor (*t*_36_ = -1.804, *p* = 0.039; *t*-test; one-tailed). In the condition with the adult target and the minus morphed distractor (*t*_36_ = -1.454, *p* = 0.077, *t*-test; one-tailed) and in the condition with the adult target and the unmanipulated distractor (neutral baby) the differences in the RTs between low T and high T subjects reached statistical trend level (*t*_36_ = -1.673, *p* = 0.051; *t*-test; one-tailed).

**Fig 6:**
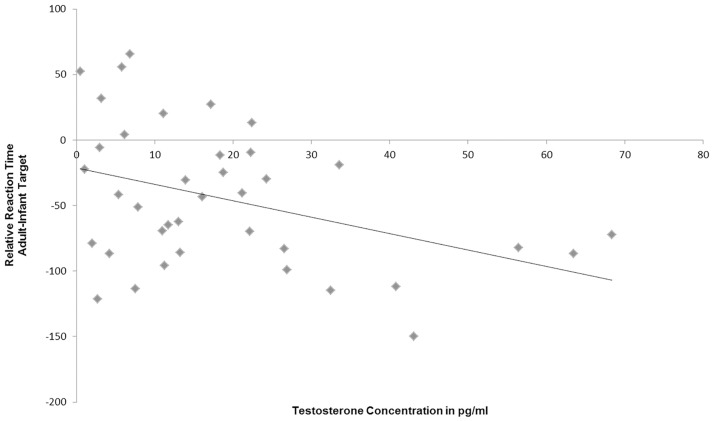
Negative correlation between salivary testosterone concentration and selective attention to human infant portraits (*r* = -0.394, *p* = 0.007; one-tailed).

As in study I, we were interested in the effects of high T concentrations on the distraction by baby schema in particular. So, we correlated T concentrations of the participants with the relative RTs (RT for adult target minus RT for baby target). Therefore we calculated the mean RT to select an adult target (i.e., the high distracting condition) and subtracted the mean RT to select an infant target (i.e. the low distracting condition). Again, we found a negative correlation between T concentration in women and the relative RTs (*r* = -0.394, *p* = 0.007, *N* = 38) (see Fig 6).

We also calculated the correlation between the T concentration of the participants and the distraction by baby schema for all three baby schema conditions separately. We found negative correlations between the habitual T concentration of the participants and the relative RTs in all three conditions (RT for adult target out of three minus, out of three neutral or out of three plus morphed babies minus RT for minus, neutral or plus morphed baby out of three adults) (high baby schema condition *r* = -0.288, *p* = 0.04, unmanipulated baby schema condition *r* = -0.345, *p* = 0.017 and low baby schema condition *r* = -0.295, *p* = 0.036) (all *p* values are reported for one-tailed tests).

## Discussion

The current study investigated the connection between endogenous testosterone and selective attention to infant faces in women. Additionally, we assessed influences of exogenous oxytocin on attentional processes regarding baby schema. Most importantly, high testosterone concentrations in female participants were negatively associated with attention towards infant faces. Moreover, our preliminary findings indicate that oxytocin might diminish the negative effects of high testosterone levels in women. Selective attention in women with high testosterone concentrations aligned to the low testosterone group after oxytocin treatment.

In our first study women located infant targets of non-threatening stimuli more quickly than the adult targets of the same species. Both, target age (adult or infant) and target species, had an effect on reaction time with participants locating infant targets faster than adult targets when pictures of humans, other primates or of other non-threatening species were presented (see Fig 3). Our results are consistent with those reported by Golle and colleagues [40] who compared the perceptual properties of human infant faces and puppy dogs in a visual adaption paradigm. They found that facial cuteness adaption transferred across both species.

Interestingly, adult predators were identified faster than infant predators (lion and polar bear, see Fig 3). This is consistent with the view that threatening stimuli have a processing advantage in the attentional system, because of their immediate relevance for survival [44]. Lions and polar bears still belong to the most dangerous terrestrial predators and represent danger in the wild [54,55]. However, the attentional benefit is remarkable because Europeans normally won’t get in contact with these predators. In conclusion, it could be a case of an inherent attention benefit, as in the case of snakes [44]. According to theory, key stimuli should trigger behavior that is beneficial for reproduction and survival [28,56]. The survival of the offspring of humans and other non-human mammals fully depends on the caregiving of their parents [30]. Since a parent’s inattentiveness could cost the offspring’s life (for example through predators or accidents), prioritized attention to newborns and infants could be adaptive to increase genetic success and thus indirectly increase parental fitness. In 95% of mammals, females are essentially responsible for rearing the offspring [57]. Cárdenas and colleagues [42] found that women took longer time and looked more often at unknown infant faces, than at unknown adults. The authors explained this as an adaptation of human cognition to infant care as a result of alloparental care in humans. Our findings thus support the notion that highly relevant stimuli should automatically and rapidly capture prioritized attention [44]. In addition, they may also conform to the view that typical child characteristic features, which elicit maternal care, could enhance survival rate of the offspring and thereby increase reproduction success, supposedly through the prioritization of attention. Furthermore, the present results are consistent with several previous studies that reported preferences for cute infant faces independent of species or ethnicity [37,39,40].

To investigate the influences of the different factors on the RTs we performed a 2 x 2 x 2 x 3 ANOVA with the factors drug group, median split of the testosterone concentration, target age and condition of the distractor. We found significant effects in the factor target age and condition of the distractor on the RTs. As the influence of the factor condition alone is hard to interpret (because it remains unclear whether the distraction or the attention is responsible for the RT differences) the significant interaction between the target age and the condition may be more meaningful.

For further exploration, we performed two separate ANOVAs for the high and the low T groups with the drug group as between subject factor. We found a main effect of the drug group only in the high T group. This might indicate that oxytocin opposes the negative effects of testosterone in the context of face processing. The neuropeptide oxytocin supports behavior that is advantageous for parental care (for example grooming and attachment; [58,59] (for review see [24]), while testosterone tends to be associated with antisocial behavior [9] (for review see [27]). In the present project, we show for the first time that endogenous testosterone in women may reduce selective attention to pictures of infants (see Figs 4 and 6), while oxytocin administration might reverse the negative effects of high testosterone levels and might probably improve face discrimination (see Fig 5). Several authors have already shown that testosterone is associated with aggressive and egocentric behavior [9,60]. This effect may be useful in the context of competition for food and territory [61], but it is not very beneficial for caretaking behavior in parent-infant interactions. It has been repeatedly demonstrated that testosterone concentration decreased during parenthood, possibly as a consequence of increased parental investment [18,19,23,62]. Only two studies have so far assessed the effect of testosterone on behaviors in the context of maternal caretaking. An administration study presented results suggesting that testosterone might upregulate neural responses to infant crying in young women. However, it remains unclear whether testosterone or its metabolite estradiol is responsible for these results [63]. Hahn and colleagues [36] investigated the effects of high salivary testosterone levels on cuteness perception. High habitual testosterone concentrations seemed to increase the time participants voluntarily decided to view cute baby faces. In this cuteness perception task, women could decide to extend the time to look at a cute baby via key press. This could explain divergent findings, because we investigated selective attention towards baby schema in a rapid attentional reaction time task. So our task was more sensitive for aspects of automatic attentional orienting, while the task used by Hahn et al. [36] was rather characterized by evaluative components of cuteness perception, which could explain the divergent findings in both studies.

### Hormones and attention towards infant faces

Salivary testosterone was analyzed to test for a correlation between the habitual testosterone status and women’s selective attention towards pictures of infants. As expected, women with higher salivary testosterone concentrations were significantly slower in orienting their attention and responding to infant targets when these were presented in the context of adult distractors. In study I, a negative correlation between salivary testosterone and selective attention to babies was observed. Women with a higher testosterone concentration exhibited longer reaction times to locate the infant (see Fig 4). This finding provides strong evidence for the hypothesis that testosterone may negatively influence attention towards infants and baby schema in women. The effects of testosterone mainly manifest in behaviors, which stand antagonistic to parental care and prosocial attributes that are important for childrearing [9,24,60]. So far, several studies found negative correlations between salivary testosterone concentration and prosocial behaviors such as caretaking and helpfulness ([9,11,12] (for review see [26]). Assuming that testosterone reduces parental care [18,19,62] it also seems plausible that it may affect the attentional system by modulating responses to baby schema in infants.

In contrast to testosterone, the „birth hormone”oxytocin has been demonstrated to promote parental care [7,46] (for review see [3,26]). To investigate the interaction between testosterone and oxytocin, we designed a second paradigm in which only human pictures were shown. In this paradigm, we manipulated the cuteness of the baby by increasing or decreasing the amount of baby schema (see [33] for a similar procedure) to investigate processing assets and drawbacks in the attentional system for babies with high or low baby schema. We expected that an increased baby schema, as a key stimulus, should automatically draw selective attention while decreased baby schema should reduce attention to these stimuli because of a greater similarity to adult individuals and less cuteness. Our data revealed that women with higher testosterone concentrations needed significantly more time to select the infant target out of three adults relative to the adult target out of three infants (see Fig 6). Previous comparison studies have shown that infants with a high baby schema were perceived to be cuter than infants with a lower baby schema [33,64]. Besides that, Glocker et al. [29] investigated the associated neural correlates. They observed an increase of activation in the nucleus accumbens with increasing baby schema (i.e., low < neutral < high BS). This area belongs to the reward system and probably has a high oxytocin receptor density in social mammals [65]. Yet, the nucleus accumbens may also be responsive to salient stimuli per se (e.g. [66]), which could lead to prioritized processing of these stimuli. One may therefore speculate that in the present study oxytocin administration diminished the negative effects of high testosterone concentrations through modulation of responses in the nucleus accumbens.

Reaction times did not differ between the oxytocin or placebo treatment groups in low testosterone women, but reaction times selectively decreased in high testosterone women in the oxytocin treatment group (see Fig 5). Although little is known about the precise interplay of testosterone and oxytocin in the brain, previous evidence indicates that they may have contrasting effects on behavior and are both associated with a change of parental investment [18,19,23,53,59,62]. Decreased testosterone concentrations during fatherhood are thought to promote caretaking behavior like affectionate touch, duration of parental vocalization and gaze to the infant’s body [18,23]. In contrast, enhanced oxytocin is associated with parenting and bonding behavior in humans and different non-human mammals [53]. Interestingly, Weisman and colleagues [23] found that oxytocin administration induced short-term increase of testosterone levels in fathers but also increased the quality of playing with the toddlers. This observation is in accordance with Frayne and Nicholson [67], who demonstrated that testosterone production increased in isolated Leydig cells from male Wistar rats that were incubated with oxytocin. Since testosterone has different effects on behavior in men and women (for review see [26]), it is possible that oxytocin modulates testosterone concentration in a different way in women. A recently published study indicates that oxytocin receptor genotype interacts with 2D:4D ratio (a biomarker to measure prenatal testosterone) in the performance of a common empathy test, but only in men, which could be another hint for a sexually dimorphic endocrine system [21].

Additionally, in females the majority of the testosterone is converted into estradiol via aromatase, whereas males have far lower estradiol concentrations [68].

However, as could be demonstrated by Weisman and colleagues [23] one does not exclude the other: Although testosterone decreased in fathers that spend more time with their children, as shown in several studies [18,23], oxytocin related increase of testosterone levels of these fathers positively correlated with parental behavior. The authors presume a more sensitive and responsive testosterone system in fathers with low testosterone concentrations. In comparison to the habitual testosterone concentration of men in general, fathers have a testosterone deficiency. This contrasts with our study, since the female participants in the present study were all nulliparous.

Furthermore, oxytocin is known to improve face processing [69–71], which might explain the decreased reaction times in the oxytocin treatment group. Please note, however, that this effect was found only in the high testosterone group. Thus, our results provide evidence for an interaction between both hormones regarding face discrimination. Previous research in the context of autism and Asperger Syndrome fits well to these data. The “Reading the Mind in the Eyes” test (RTME–test) helps to measure perspective taking abilities in humans. Testosterone administration impaired the performance in the RTME-test depending on the prenatal androgen marker (2D:4D ratio) indicating that testosterone administration led to significant impairment of cognitive empathy in women with high prenatal level of androgens, but not in women with low prenatal androgen levels [15]. Hence, it is possible that androgens have a negative influence on face reading and processing. In contrast, oxytocin administration not only improved the cognitive empathy in the RTME-Test in men without autism or Asperger Syndrome [7] but also increased activation in brain areas during social information processing in children with autism [72]. According to our results it is highly likely that both hormones modulate social cognition in an opposing fashion.

Beyond expectation, in the second study the reaction time differences between the low, neutral (unmanipulated) and high baby schema and the adult target condition did not correspond to our assumption that baby faces should be faster identified (see Fig 5). A possible explanation for this could be that the student group that we tested had only little experience with babies, so that the participants were more familiar or even attracted by same age stimuli. This could also be one reason for the low relative RTs in the second study (RTs for infant targets were longer than RTs for adult targets with infant distractors (Fig 6)). We suppose that the young female participants were also interested in same age stimuli. But nonetheless, the relative RTs of the participants decreased with higher testosterone concentrations. The attention towards infant faces was reduced in women with higher T concentrations—as in Study I.

Still, examining the present research questions with participants potentially more sensitive to baby schema (e.g., mothers compared with nulliparous) and to realize the target detection with older adult stimuli (that do not represent a potential partner or competitor) could be one of several possible avenues for future studies in this regard.

## Conclusion

Given the findings of the present project, there is strong evidence that endogenous testosterone negatively modulates selective attention to infant faces. Repeatedly, we found a negative correlation between the distraction by infant faces and the habitual testosterone concentration of female participants. In contrast to the negative effects of endogenous testosterone, exogenous oxytocin apparently seems to promote face discrimination ability, but only in women with high endogenous testosterone levels. These results were not limited to infant faces but also to adults. A possible explanation could be, that the oxytocin administration also promoted the attraction to the same age adult stimuli as described in the literature [73]. On the whole, the results of the present project provide initial support for the idea of a complex interplay of testosterone and oxytocin in the modulation of social behavior and maternal care. Based on the small sample size further research is needed.

## Supporting Information

S1 Table Behavioral Data Study 1. (PDF)

S2 Table Behavioral Data Study 2. (PDF)
