# Supplementary material for: Endogenous Testosterone and Exogenous Oxytocin Modulate Attentional Processing of Infant Faces
Source: PLoS One. 2016 Nov 18;11(11):e0166617. doi: 10.1371/journal.pone.0166617 (PMC5115757; doi:10.1371/journal.pone.0166617)
Supplement: S1 Table — (PDF) [file pone.0166617.s001.pdf]

S1 Table. Behavioral Data Study 1.

Reactiontime_Data (ms)

ID	T_conc.	Target_adult_polar_bear	Target_baby_polar_bear	Target_adult_dog	Target_baby_dog	Target_adult_lion	Target_baby_lion
1.00	20.16	1326.28	1534.85	1591.10	1477.53	1159.24	1241.11
2.00	22.98	1434.13	1448.67	1498.55	1608.37	1246.36	1305.15
4.00	32.77	1552.28	1674.44	1704.55	1708.38	1450.68	1496.10
5.00	12.29	1474.83	1443.60	1595.10	1472.26	1300.01	1316.29
6.00	2.52	1684.39	1731.69	1734.41	1701.56	1403.77	1439.37
7.00	7.04	1619.07	1473.18	1630.41	1591.46	1280.47	1476.37
8.00	16.41	1238.39	1479.68	1426.75	1525.68	1225.16	1454.14
9.00	9.58	1514.79	1622.20	1424.31		1364.33	1583.61
10.00	13.97	1435.56	1379.03	1549.87	1471.44	1370.31	1358.15
11.00	2.08	1590.59	1794.60	1796.52	1814.97	1566.02	1658.98
12.00	15.02	1348.03	1263.89	1493.83	1343.91	1296.68	1166.82
14.00	9.24	1230.91	1561.52	1309.08	1332.13	1238.79	1170.60
15.00	25.96	1420.69	1569.40	1507.15	1574.03	1404.30	1487.56
17.00	38.13	1302.83	1492.55	1565.25	1612.06	1139.63	1505.47
18.00	16.25	1536.37	1543.09	1650.49	1150.60	1195.57	1429.68
19.00	13.24	1576.26	1634.09	1634.39	1597.29	1398.88	1469.84
20.00	7.86	1632.86	1690.87	1753.85	1736.95	1483.68	1577.54
21.00	13.11	1566.17	1576.25	1703.30	1682.78	1327.50	1537.27
22.00	13.23	1584.24	1681.33	1722.84	1786.16	1507.67	1689.24
24.00	23.57	1508.53	1503.86	1566.43	1434.90	1348.82	1428.53

ID	Target_adult_woman	Target_baby_woman	Target_adult_man	Target_baby_man	Target_adult_orangutan	Target_baby_orangutan
1.00	1057.80	889.16	967.67	791.67	1334.82	1288.14
2.00	1178.15	1085.81	1048.66	861.67	1295.47	1283.55
4.00	1162.20	1234.20	1042.92	1097.89	1344.67	1431.80
5.00	1114.68	971.76	867.24	793.60	1279.54	1191.54
6.00	1294.85	1026.29	1195.56	936.79	1643.56	1388.50
7.00	1141.67	1017.95	1140.84	861.85	1352.03	1325.28
8.00	1095.21	1130.96	967.50	962.94	1545.98	1525.34
9.00	1376.20	1133.71	1260.45	1066.13	1545.50	1546.65
10.00	1053.62	1024.34	853.54	769.66	1201.04	1184.06
11.00	1430.63	1430.60	1282.80	1244.73	1548.49	1658.53
12.00	1058.73	1000.27	924.53	851.96	1451.46	1180.29
14.00	1024.86	1005.73	894.58	920.89	1289.31	1175.77
15.00	1249.27	1235.19	1055.14	1074.93	1487.14	1490.08
17.00	1109.17	1076.05	1015.10	940.57	1266.80	1335.45
18.00	1291.42	942.13	1007.84	846.37	1507.58	1433.93
19.00	1135.09	1081.01	1004.77	938.52	1553.02	1433.38
20.00	1397.88	1287.81	1257.86	1111.13	1512.46	1603.80
21.00	1222.10	1096.11	1126.41	982.72	1545.09	1355.92
22.00	1399.47	1284.34	1140.92	1129.16	1614.94	1633.16
24.00	1113.40	1051.55	985.86	905.30	1333.46	1338.97

ID	Target_adult_goat	Target_baby_goat	Delta_polar_bear	Delta_dog	Delta_lion	Delta_human_female	Delta_human_male	Delta_orangutan
1.00	1380.42	1354.82	-208.57	113.57	-81.87	168.65	176.00	46.68
2.00	1402.11	1287.54	-14.53	-109.82	-58.79	92.34	186.99	11.93
4.00	1598.02	1598.94	-122.16	-3.83	-45.42	-72.00	-54.97	-87.12
5.00	1548.50	1355.38	31.24	122.84	-16.27	142.92	73.64	87.99
6.00	1690.47	1629.88	-47.31	32.85	-35.61	268.56	258.77	255.06
7.00	1434.40	1451.21	145.89	38.95	-195.90	123.71	278.99	26.75
8.00	1388.89	1467.13	-241.29	-98.93	-228.99	-35.75	4.56	20.64
9.00	1432.91	1517.13	-107.41		-219.28	242.50	194.32	-1.16
10.00	1334.63	1233.50	56.54	78.43	12.16	29.28	83.89	16.98
11.00	1799.66	1663.89	-204.01	-18.45	-92.96	0.03	38.07	-110.03
12.00	1432.82	1248.86	84.14	149.91	129.86	58.47	72.57	271.18
14.00	1352.34	1200.48	-330.61	-23.05	68.19	19.13	-26.30	113.54
15.00	1505.74	1493.62	-148.71	-66.89	-83.26	14.07	-19.79	-2.94
17.00	1528.63	1501.61	-189.72	-46.81	-365.85	33.12	74.53	-68.65
18.00	1436.48	1386.23	-6.72	499.89	-234.11	349.29	161.47	73.65
19.00	1528.63	1463.98	-57.83	37.10	-70.96	54.08	66.25	119.64
20.00	1666.53	1576.26	-58.01	16.90	-93.86	110.07	146.74	-91.35
21.00	1616.20	1582.38	-10.08	20.52	-209.77	126.00	143.69	189.17
22.00	1639.48	1602.91	-97.09	-63.33	-181.57	115.13	11.75	-18.23
24.00	1538.85	1495.60	4.66	131.53	-79.71	61.85	80.56	-5.51

ID	Delta_goat	Delta_all
1.00	25.60	34.29
2.00	114.57	31.81
4.00	-0.93	-55.20
5.00	193.13	90.78
6.00	60.59	113.27
7.00	-16.80	57.37
8.00	-78.24	-94.00
9.00	-84.22	93.63
10.00	101.12	54.05
11.00	135.77	-35.94
12.00	183.96	135.73
14.00	151.86	-3.89
15.00	12.12	-42.20
17.00	27.02	-76.62
18.00	50.26	127.68
19.00	64.65	30.42
20.00	90.27	17.25
21.00	33.82	41.91
22.00	36.57	-28.11
24.00	43.25	33.81
