# Supplementary material for: Endogenous Testosterone and Exogenous Oxytocin Modulate Attentional Processing of Infant Faces
Source: PLoS One. 2016 Nov 18;11(11):e0166617. doi: 10.1371/journal.pone.0166617 (PMC5115757; doi:10.1371/journal.pone.0166617)
Supplement: S2 Table — (PDF) [file pone.0166617.s002.pdf]

S2 Tabel. Behavioral Data Study 2.

Reactiontime_Data (ms)

ID	T_conc.	Instruktion	Medikation	Distractor_Minus	Distractor_Neutral	Distractor_Plus	Target_Minus
3	2.69	placebo	placebo	1024.13	968.05	1009.99	1108.88
4	26.53	placebo	oxytocin	1186.28	1221.51	1175.05	1172.47
5	11.73	oxytocin	placebo	1159.11	1163.46	1183.55	1249.63
6	6.86	oxytocin	oxytocin	1385.87	1315.52	1266.73	1292.62
10	3.20	placebo	oxytocin	859.19	901.71	825.68	829.72
11	63.48	oxytocin	placebo	1102.58	1095.24	1114.12	1164.54
13	24.30	placebo	placebo	1286.74	1227.98	1139.75	1263.60
14	10.99	placebo	placebo	1168.25	1157.27	1142.78	1216.63
18	22.42	oxytocin	placebo	1253.20	1141.53	1182.10	1195.36
19	22.27	oxytocin	placebo	1142.73	1107.10	1057.35	1150.35
20	40.79	placebo	placebo	1186.01	1155.71	1120.92	1269.95
21	5.82	placebo	oxytocin	989.13	1012.14	982.20	922.06
22	2.01	placebo	oxytocin	909.38	910.73	947.13	1018.10
23	5.44	oxytocin	placebo	1182.67	1123.03	1103.50	1196.93
24	2.97	placebo	placebo	1016.40	988.75	992.98	999.65
25	16.07	placebo	placebo	1338.80	1278.85	1262.08	1326.84
26	.53	placebo	placebo	1159.53	1091.48	1067.55	1079.18
27	11.09	oxytocin	oxytocin	1277.99	1290.66	1292.40	1219.09
28	56.47	oxytocin	placebo	1256.89	1200.21	1238.55	1242.79
29	18.78	placebo	oxytocin	975.45	956.92	1016.35	1057.93
30	1.05	oxytocin	oxytocin	1273.61	1287.66	1273.03	1331.00
33	18.34	placebo	placebo	1382.52	1386.99	1430.30	1430.87
38	17.17	oxytocin	oxytocin	1181.90	1172.17	1116.50	1154.25
41	26.89	oxytocin	oxytocin	1025.88	1064.35	1166.61	1177.99
42	6.14	oxytocin	oxytocin	1089.90	1069.81	1013.90	1040.45
43	43.07	placebo	oxytocin	1064.25	1042.54	994.38	1220.73
45	21.21	oxytocin	placebo	1116.13	1080.10	1097.88	1111.88
46	13.23	placebo	oxytocin	1043.11	1039.75	932.85	1053.40
50	22.16	oxytocin	placebo	1193.40	1183.90	1139.68	1256.63
52	68.38	oxytocin	oxytocin	1204.10	1169.11	1154.28	1265.53
53	33.61	oxytocin	placebo	1226.90	1182.89	1138.90	1189.16
55	11.23	oxytocin	oxytocin	1207.35	1219.22	1140.38	1323.24
56	13.02	oxytocin	oxytocin	1092.97	982.35	1028.64	1080.34
57	7.57	placebo	oxytocin	1126.37	1044.98	1078.02	1104.91
58	7.92	placebo	placebo	1229.26	1175.42	1253.43	1246.60
61	4.23	oxytocin	oxytocin	1250.47	1178.25	1153.38	1263.20
62	13.98	placebo	oxytocin	1073.38	1077.01	1105.50	1057.85
70	32.48	oxytocin	placebo	1347.04	1394.20	1352.11	1478.04

ID	Target_Neutral	Target_Plus	DeltaMinus	DeltaNeutral	DeltaPlus	Delta_all	TKonzSplit
3	1090.08	1167.39	-84.75	-122.03	-157.40	-121.39	.00
4	1356.19	1303.10	13.81	-134.67	-128.04	-82.97	1.00
5	1267.47	1182.95	-90.53	-104.01	.60	-64.64	.00
6	1263.75	1215.00	93.25	51.77	51.73	65.58	.00
10	853.44	808.47	29.47	48.27	17.21	31.65	.00
11	1195.59	1211.74	-61.96	-100.34	-97.62	-86.64	1.00
13	1240.10	1239.52	23.14	-12.11	-99.77	-29.58	1.00
14	1223.53	1236.47	-48.38	-66.26	-93.70	-69.44	.00
18	1188.61	1152.76	57.84	-47.08	29.34	13.37	1.00
19	1109.46	1075.90	-7.62	-2.36	-18.55	-9.51	1.00
20	1319.42	1209.00	-83.94	-163.71	-88.08	-111.91	1.00
21	957.19	936.99	67.06	54.95	45.21	55.74	.00
22	1025.59	959.50	-108.73	-114.87	-12.38	-78.66	.00
23	1162.51	1175.03	-14.26	-39.48	-71.53	-41.75	.00
24	1044.85	970.72	16.75	-56.10	22.25	-5.70	.00
25	1311.36	1371.93	11.96	-32.51	-109.85	-43.47	1.00
26	1029.48	1052.43	80.35	62.01	15.13	52.49	.00
27	1293.73	1287.33	58.90	-3.07	5.07	20.30	.00
28	1401.45	1298.15	14.10	-201.25	-59.60	-82.25	1.00
29	986.32	978.58	-82.48	-29.40	37.78	-24.70	1.00
30	1313.95	1256.08	-57.39	-26.29	16.95	-22.25	.00
33	1443.28	1360.78	-48.34	-56.28	69.52	-11.70	1.00
38	1116.35	1118.54	27.65	55.82	-2.04	27.15	1.00
41	1190.85	1185.40	-152.11	-126.50	-18.79	-99.13	1.00
42	1068.85	1052.65	49.45	.96	-38.75	3.89	.00
43	1157.71	1171.73	-156.48	-115.17	-177.34	-149.66	1.00
45	1168.93	1134.30	4.25	-88.82	-36.43	-40.33	1.00
46	1109.49	1110.79	-10.29	-69.74	-177.94	-85.99	.00
50	1244.85	1225.23	-63.22	-60.95	-85.55	-69.91	1.00
52	1273.61	1205.18	-61.43	-104.51	-50.89	-72.28	1.00
53	1239.23	1177.83	37.74	-56.34	-38.92	-19.17	1.00
55	1270.61	1260.18	-115.89	-51.40	-119.80	-95.69	.00
56	1123.25	1086.86	12.63	-140.90	-58.22	-62.16	.00
57	1262.92	1222.70	21.46	-217.94	-144.68	-113.72	.00
58	1313.43	1251.13	-17.34	-138.01	2.29	-51.02	.00
61	1354.45	1224.47	-12.73	-176.20	-71.09	-86.67	.00
62	1192.13	1098.20	15.53	-115.11	7.30	-30.76	1.00
70	1466.26	1493.41	-131.00	-72.06	-141.30	-114.79	1.00
